# A Putative PP2C-Encoding Gene Negatively Regulates ABA Signaling in *Populus euphratica*

**DOI:** 10.1371/journal.pone.0139466

**Published:** 2015-10-02

**Authors:** Jinhuan Chen, Dongzhi Zhang, Chong Zhang, Xinli Xia, Weilun Yin, Qianqian Tian

**Affiliations:** 1 College of Biological Sciences and technology, Beijing Forestry University, Beijing, China; 2 National Engineering Laboratory for Tree Breeding, Beijing Forestry University, Beijing, China; Chinese Academy of Sciences, CHINA

## Abstract

A PP2C homolog gene was cloned from the drought-treated cDNA library of *Populus euphratica*. Multiple sequence alignment analysis suggested that the gene is a potential ortholog of HAB1. The expression of this *HAB1* ortholog (*PeHAB1*) was markedly induced by drought and moderately induced by ABA. To characterize its function in ABA signaling, we generated transgenic *Arabidopsis thaliana* plants overexpressing this gene. Transgenic lines exhibited reduced responses to exogenous ABA and reduced tolerance to drought compared to wide-type lines. Yeast two-hybrid analyses indicated that PeHAB1 could interact with the ABA receptor PYL4 in an ABA-independent manner. Taken together; these results indicated that PeHAB1 is a new negative regulator of ABA responses in poplar.

## Introduction

The phytohormone abscisic acid (ABA) plays a key role in different plant developmental processes as well as in the perception of abiotic stresses such as drought, salt and cold [1,2]. ABA is well established as a mediator of physiological responses to water deficit. Water stress can lead to an increase of ABA level in plants, resulting in various adaptive responses, including stomatal closure and gene expression [3,4]. The ABA signal transduction system involves a complex network of both positive and negative regulators. Previous reports reviewed Clade A PP2C/Protein Phosphatase 2C suppressed the release of signaling triggered by ABA [5,6]. In *Arabidopsis*, *PP2CA*, *ABI1* and *ABI2* were characterized as key negative regulators of ABA signaling [7]. Studies on loss-of-function alleles of *ABI1* and *ABI2* and the generation of double revealed that *ABI1* and *ABI2* function partially redundant as inhibitors of ABA signaling [8]. Experimental data also alternatively support that PP2C-like gene could act as a positive regulator rather than a negative regulator of ABA signaling [9], which indicated that PP2C play complicated roles in plants. ABA-related Clade A PP2Cs triggered the regulation of numerous processes by interaction with multiple proteins. Recently, crystal graphic studies revealed the complex structure of PP2C-ABA-PYR1/PYL/RCAR (Pyrabactin Resistance 1 /PYR1-Like /Regulatory component of ABA receptor), among which PYL was identified as an intracellular ABA receptor [10–16]. Ongoing researches also demonstrates that PYR/PYL/RCAR receptors have shown preferences in substrate specificity and selectively inhibit specific PP2Cs [17]. These studies triggered considerable interest in ABA signaling in different plant species.

As the second published genome of poplar species [18], *Populus euphratica* is a famous poplar species that is mainly distributed in deserts and noted for its high abiotic stress tolerance. However, to our knowledge, only one *PP2C* member has been characterized in poplar [19], and few studies have focused on interactions between type 2C protein phosphatases and putative ABA receptors in poplar. To test whether *PP2C* member may be crucial for ABA response in *P*. *euphratica* and whether it could function through PP2C-PYL interaction, we cloned a potential *HAB1* ortholog gene from *P*. *euphratica* and generated *Arabidopsis thaliana* transgenic lines to characterize the function of this gene in ABA signaling. An attempt also has been made to identify the interacting protein of this *HAB1* ortholog gene by screening a cDNA library to illustrate its potential function.

## Results

### *PeHAB1* identification

**Fig 1:**
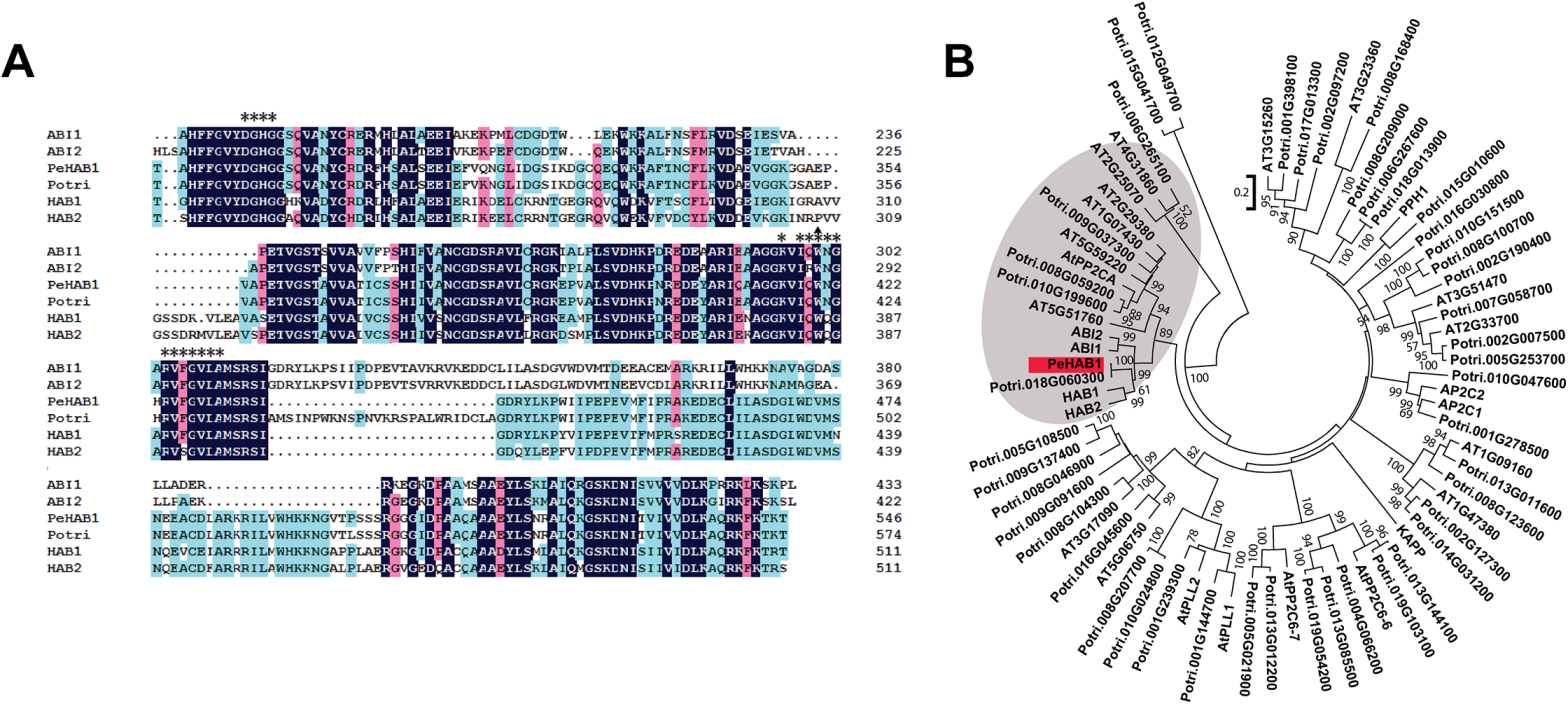
Sequence analysis of PeHAB1. (A) Sequence alignment of the deduced amino acid sequence of PeHAB1 with four Clade A *Arabidopsis* PP2C members including AtABI1, AtABI2, AtHAB1, AtHAB2, and one *Populus trichocarpa* gene Potri.018G060300. Identical amino acid residues are highlighted in black, while conserved amino acid residues are indicated by colored shading. Asterisk codes indicated the amino acid residues involved in interaction with PYLs. Trp420 was marked with a small triangle. (B) Phylogenetic tree of PeHAB1, 27 representative PP2C members from *Arabidopsis*, and 45 PP2C candidates from *P*. *trichocarpa*. The ABA-related Clade A PP2Cs were indicated by the grey shade. The tree was constructed by Neighbor–Joining method based on multiple alignments of full-length amino acid sequences with bootstrap values of 1000 replicates.

To study the response of drought, a cDNA library was constructed by using leaves of *P*. *eupharatica* as the source of mRNA. The titers of primary cDNA library and amplified library were 2.2×10^6^ and 1.2×10^10^, respectively, and the recombinant was >87%. Over 1500 clones were randomly sequenced. The inserted fragments ranged from 500 bp to 3000 bp. Among the fragments, a putative PP2C member has been screened as full length for five times. According to the BLAST searching method, this sequence was supposed to encode a Clade A PP2C family member and was given a temporary name *PeHAB1*. Full-length *PeHAB1* was then isolated from cDNAs of roots, leaves and stems, indicating that *PeHAB1* could be expressed in all these organs. The isolated cDNA fragment contains 1641 bp and encodes a protein with 546 amino acids (Genebank ID: KP055179), with a calculated molecular mass of 58.79 kD and a predicted pI of 4.47. The overall sequence similarity between AtHAB1 and PeHAB1 is 45.08% (Fig 1A). This percentage is reasonable, considering that PP2C did not share similarities in the C-terminal part with their paralogs or orthologs [20]. Certain motifs, such as the PYL interaction site, are highly conserved in PeHAB1 (Fig 1A). We further compiled an alignment that included *PeHAB1*, 45 annotated PP2C candidates from poplar genome, and 27 representative *Arabidopsis PP2Cs*, and then constructed a phylogenetic tree. As expected, *PeHAB1* was clustered in Clade A with a position close to *HAB1* (Fig 1B). Therefore, the *P*. *euphratica* gene was designated as *PeHAB1*.

### Expression pattern of four annotated ABA-related PP2Cs from *Populus tricocarpa*

**Fig 2:**
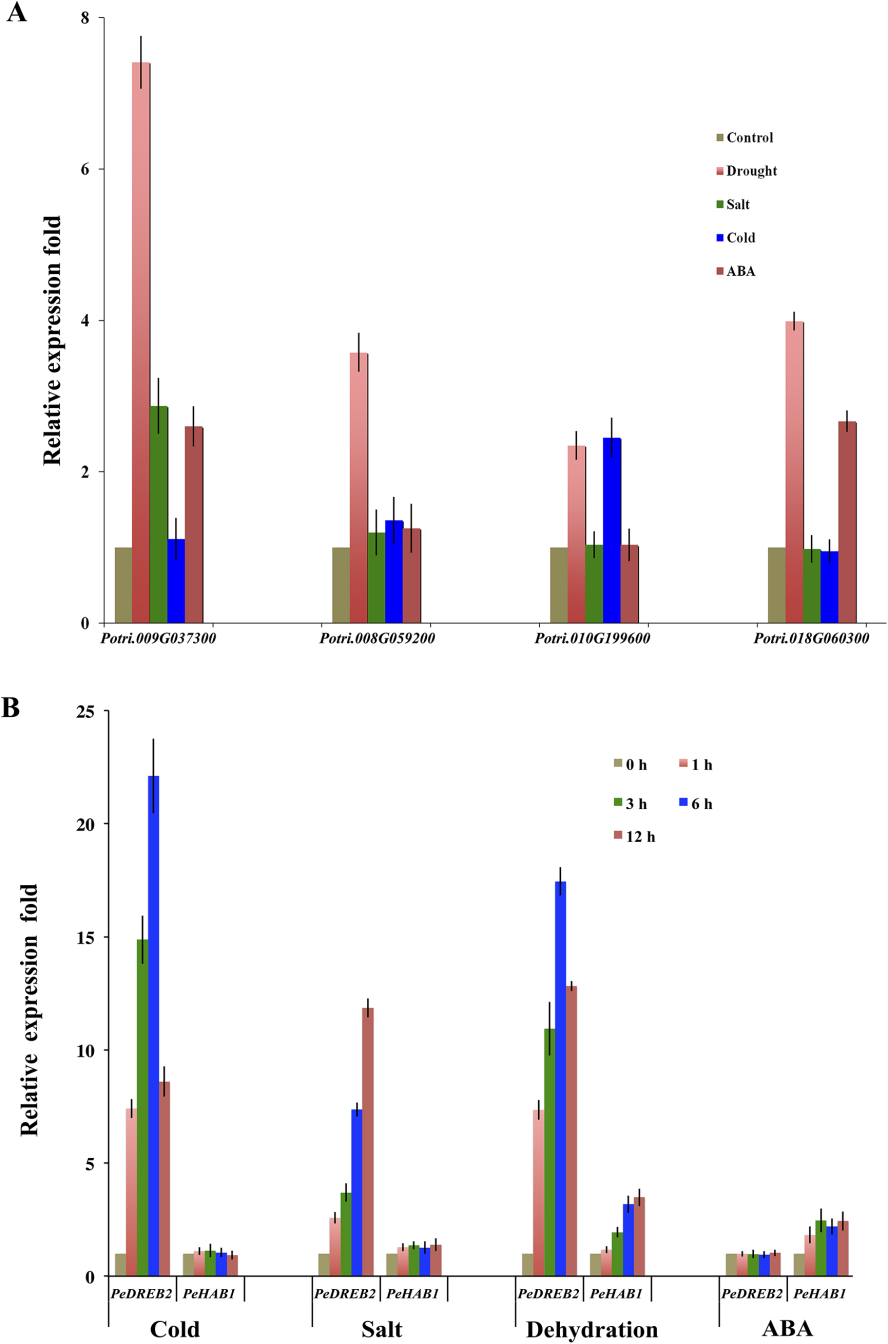
Real-time PCR analysis of several Clade A PtPP2Cs and *PeHAB1* under ABA, NaCl, drought, and cold treatment. (A) Relative gene expression analysis of four Clade A PtPP2Cs under various abiotic stress treatments. (B) Quantitative RT-PCR was performed with *PeActin* as an internal reference and *PeDREB2* as an experimental control. The expression level in untreated leaves was assigned a value of 1. The capital characters represent 200 μM ABA (ABA), 300 mM NaCl (S), dehydration (D), and 4°C cold, respectively. Data are presented as means ± SE from three independent biological replicates.

Our qPCR analysis indicated that all the Clade A *PtPP2C* candidates were differentially expressed in response to various abiotic stress treatments. In general, all four genes involved in analysis were induced by drought treatment, and the highest expression level was observed in Potri.009G037300. Besides drought treatment, Potri.009G037300 also showed a high induction under salt treatment, and Potri.010G199600 was seen to be accumulated under cold treatment. The accumulation of Potri.018G060300 increased after both drought and ABA treatments, whereas Potri.008G059200 was the member whose expression was alerted only after drought treatment (Fig 2A).

### *PeHAB1* is induced by dehydration and ABA

The expression level of *PeHAB1* under different abiotic stresses was determined by quantitative real-time PCR analyses, with *PeActin* as a housekeeping gene. A multiple-abiotic-stress- inducible gene, *PeDREB2*, was introduced in real-time PCR analysis as an experimental control [21]. The results indicated that *PeHAB1* accumulates its transcripts after ABA and drought treatments, with a relative higher accumulation under drought treatment (Fig 2B). The expression of *PeHAB1* was not significantly stimulated by high salt and cold stresses. Gene expression patterns often illustrate the gene function. Thus, our results suggested that *PeHAB1* may participate in ABA or drought responses.

### PeHAB1 interacts with abscisic acid receptor PePYL4

**Fig 3:**
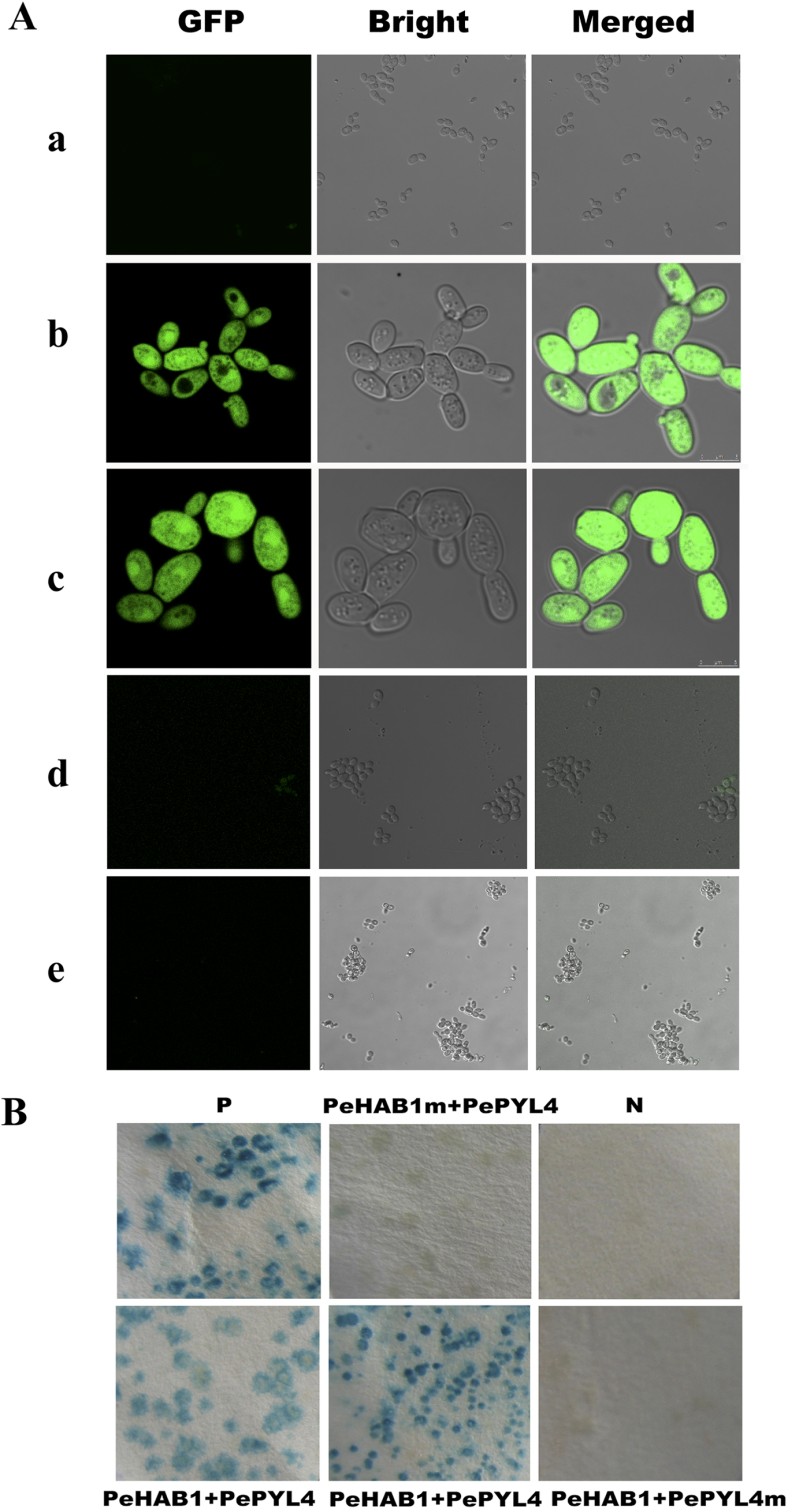
Yeast two-hybrid assay for PeHAB1- interacting proteins. (A) Yeast two-hybrid interaction test of PeHAB1 with PePYL4. Interactions were detected by growth of yeast on selective medium and the detection of a GFP signal. (a-d) indicated negative control; yeast harbored PeHAB1 and PePYL4; Supplied interacting pairs that served as positive control; yeast harbored PePYL4 and PeHAB1 of substitution mutation W420A and yeast harbored PeHAB1 and PePYL4 of substitution mutation P124S. (B) *β*-galactosidase activity (blue) in streaks of yeast cells on a culture plate as readouts of protein-protein interactions by using the yeast two-hybrid system.

A new yeast-two-hybrid method, with PeHAB1 protein as bait, was applied to search the potential PeHAB1-interacting proteins in *P*. *euphratica*. After transformation with the prey cDNA library of *P*. *euphratica*, dozens of putative clones were screened from the total of 1 × 10^6^ recombinant colonies. To discriminate true positive clones from spontaneous yeast mutations, we retransformed the yeast reporter strain with the cDNA of each potential candidate. Finally, five positives clones encoding the same gene were retrieved. The isolated cDNA encompassed an open reading frame (ORF) of 666 bp with a predicted protein of 221 amino acids (Genebank ID: KP064303, S1A Fig). A BLAST search against the NCBI database indicated that the cDNA belongs to the RCAR/PYR/PYL gene family. It shares 98% identity with Potri.006G104100 from *P*. *trichocarpa*, 82% identity of KDP22835.1 from *Jatropha curcas*, and 65% identity of AtPYL4 from *Arabidopsis* at the amino acid level. The gene contains conserved gate region and latch region, which suggested that the gate-latch-lock mechanism is employed in its hormone binding [22]. The phylogenic tree that was constructed by using all *Arabidopsis PYLs* indicates that its most likely orthologous gene is *PYL4* (S1B Fig). Thus, this gene was hereby named *PePYL4*. We further confirmed the interaction between PeHAB1 and PePYL4 by both MoBiTec Grow’n’Glow and GAL4 Clontech systems, and the results showed that PeHAB1 can interact with PePYL4 without ABA supplementation (Fig 3A).

Certain PP2C members have been reported to interact with PYL through the region around its active site and a flap sub-domain [22,23]. The critical active site residues of Arg199, Glu203, Asp204, Asp243, Gly244, His245, Asp432, and Asp492, which were previously reported in *Arabidopsis*, were conserved in PeHAB1 [23,24], corresponding to the residues of Arg242, Glu246, Asp247, Asp288, Gly289, His290, Asp465, and Asp527, respectively (Fig 1). Previous reports have established a critical tryptophan residue that can be bound by PYLs and/or ABA by pointing into the hydrophobic pocket of PYLs [12,22]. Thus, we introduced a mutation to the corresponding residue of Try420 in PeHAB1 by substituting Try to Ala. As a result, W420A mutate showed a remarkable reduction of affinity to PePYL4 (Fig 3). We also introduced a P124S mutation to PeHAB1, which resulted in a disability of affinity. These results indicated that PeHAB1 may interact with PePYL4 using the gate-latch-lock mechanism.

### Overexpression of *PeHAB1* reduces ABA sensitivity

**Fig 4:**
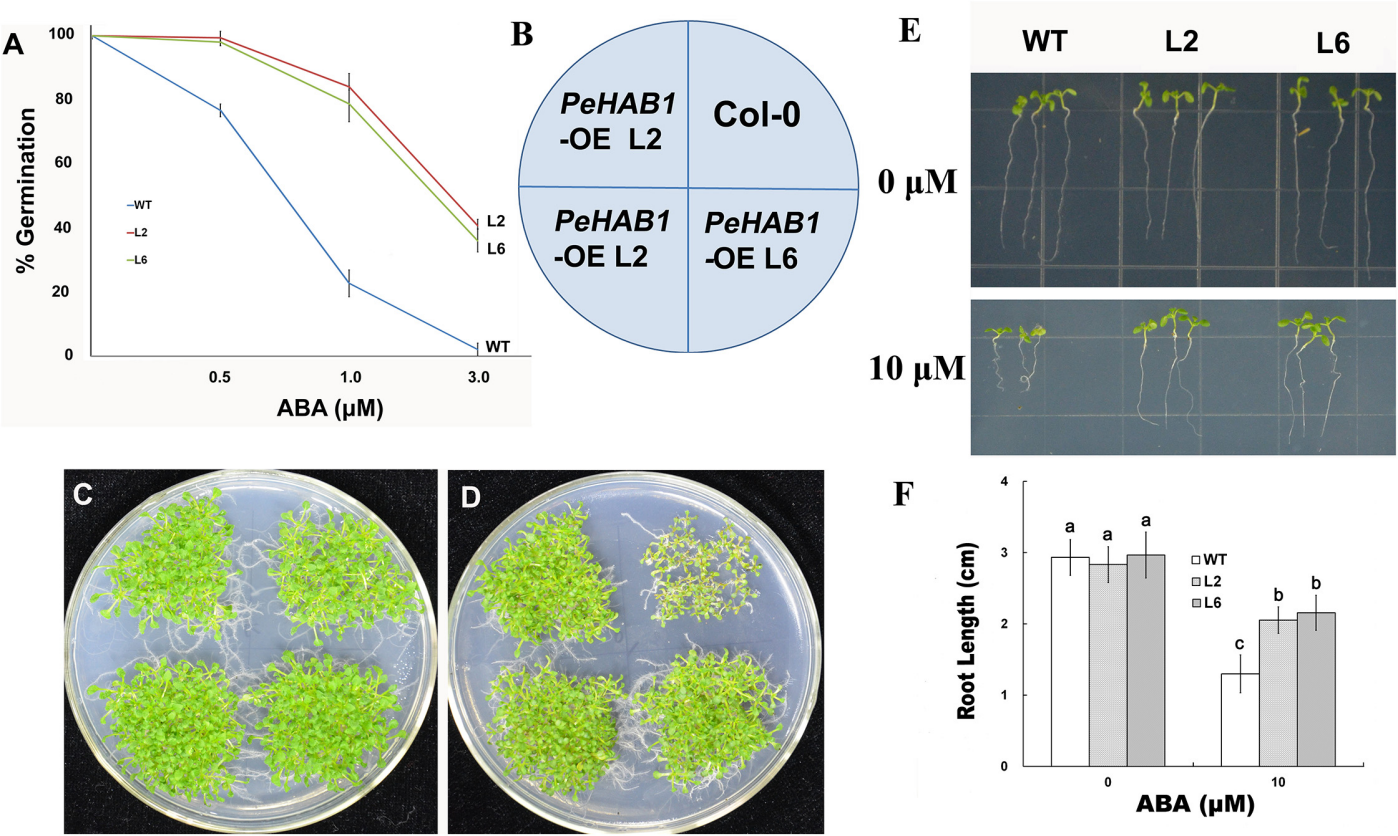
Constitutive expression of *PeHAB1*confers ABA insensitivity in transgenic plants during seed germination and root growth development. (A) The emergence rate of green cotyledons from Col-0 and *PeHAB1* transgenic seeds plated on MS medium supplemented with different concentration of ABA. Approximately 100 seeds were used in each experiment. Error bars represent the standard deviation. (B-D) Col-0 and two independent *35S*::*PeHAB1* lines were germinated and grown in medium (MS) supplemented with 0.75 μM ABA. Photographs were taken after 14 days. (E-F) Root length in MS medium supplemented with 10 μM ABA and its correspondingly statistical analysis. Data means ±SE from three independent experiments (*n* = 30 seeds).

To investigate whether sustained transcriptional overexpression of *PeHAB1* affects ABA sensitivity, we analyzed two homozygous lines with similar expression levels of *PeHAB1* by performing assays on the germination and early seeding development of ABA treated seeds. For germination assay, seeds were planted in MS medium supplemented with different concentrations of exogenous ABA. As a result, *35S*::*PeHAB1* homozygous lines showed similar germination ratios to wild-type in the absence of ABA. However, the germination ratio of wide-type plants was strongly inhibited and only less inhibitory effect was found in transgenic lines after ABA was added (Fig 4A). We also tested the germination rate and development performance at an ABA concentration of 0.75 μM. As expected, *PeHAB1* overexpressing lines showed a higher frequency of seedlings with green cotyledons and a faster growth rate (Fig 4B and 4D).

Root growth is usually considered as a standard criterion in evaluating the ABA sensitivity of plants because of the inhibitory role of ABA plays on roots [25]. To test the sensitivity of root in response to exogenous ABA, we transferred 5-day-old seedlings to solid MS media supplemented with 10 μM ABA and then incubated plates in a vertical position in a growth chamber. As a result, a significant reduction of root growth in wild-type plants was observed. However, the phenotypes of transgenic lines did not considerably change with exogenous ABA (Fig 4E and 4F), indicating that *PeHAB1* overexpression reduced the sensitivity of the root to ABA.

Overall, the overexpression of *PeHAB1* resulted in an ABA-insensitive phenotype with ABA-mediated inhibition of seedling germination and root growth. This result indicated that *PeHAB1* was a negative regulator of ABA signaling.

### Overexpression of *PeHAB1* enhances water loss of plants

**Fig 5:**
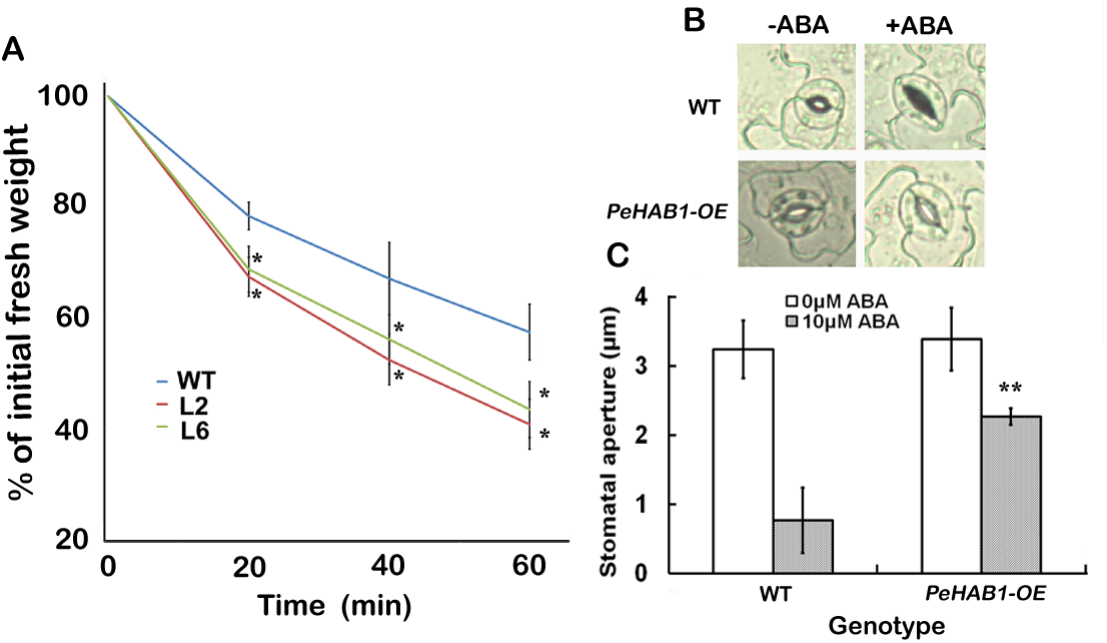
Phenotype tests for water loss and stomata opening under ABA treatment. (A) Water loss analysis. Time courses for the percentage of initial fresh weight were recorded from the detached leaves of wild-type controls, L2, and L6. Data are averages ±SE from three independent experiments (*n* = 10 plants). Asterisks indicate significant difference between transgenic lines and wild-type at different time points (* *P*<0.05; Student’s *t*-test). (B) Stomatal photographs of *35S*::*PeHAB1* transgenic lines and wild-type plants in the presence or absence of ABA. (C) Average stomatal aperture of *PeHAB1* overexpression transgenic lines and wild type plants in response to ABA. Data are averages ±SE from three independent experiments (*n* > 20 stomata). Asterisks indicate significant difference between transgenic lines and wild-type with or without ABA (** *P*<0.01; Student’s *t*-test).

Transpiration rate assays showed that detached leaves of *PeHAB1*-overexpressing plants lose water more quickly in comparison with those of wild-type plants (Fig 5A; Student’s *t*-test), indicating a reduced tolerance of transgenic plants to drought stress. To assess whether the drought tolerance reduction was related to an alteration in sensitivity to ABA, we measured the effect of ABA on stomatal aperture. The results showed a significant difference of stomatal aperture between *PeHAB1* overexpressing lines and wild-type under 10 μM ABA (Fig 5B and 5C), indicating that ABA-triggered stomatal closure is blocked in transgenic lines, which may result in an enhanced transpiration rate afterwards in *PeHAB1* overexpressing lines.

## Discussion

Notable progress has been recently made in ABA signaling by identifying the *Arabidopsis* PYR/PYL/RCAR gene family as ABA receptors that inhibit group-A PP2C activity in response to ABA. PYL-PP2C interaction has been identified in organisms, such as *Artemisia annua*, *Oryza sativa*, and soybean, by experimental analysis [25]. To explore ABA components in *P*. *euphratica*, we cloned a putative *PP2C* gene by cDNA library screening method and identified a PP2C-PYL interaction by using this PP2C to screen a yeast cDNA library.

*PeHAB1* gained its name because of its high sequence similarity to *AtHAB1*. BLAST research analysis based on the poplar database revealed more than 76 *PP2Cs* in poplar. Thus, we preferred the overexpression method instead of constructing knock-down or knock-out lines to avoid the defect of mutation analysis that may be caused by functional redundancy. In *Arabidopsis*, the phenotype of *hab1-1* plants and *HAB1*-overexpressing plants are consistent, showing the function of *HAB1* as a negative regulator of ABA signaling [26–28]. Here, ectopic overexpression of *PeHAB1* in *Arabidopsis* showed an ABA-insensitive phenotype, including reduction of the negative effects of ABA on germination and root growth, as well as the reduction of the positive effects that ABA signaling normally has on mounting appropriate responses to drought/dehydration stress. All these results indicated a negative regulator *PeHAB1* function in ABA signaling. Very recently, a predicted *PP2CA* ortholog named *G059200* was cloned and functionally characterized in poplar [29]. The performance of *G059200* transgenic lines exhibited reduced responses to exogenous ABA and reduced tolerance to salt stress, indicating that the ABA signal mediated by Clade A PP2Cs may be conserved in poplar. However, whether the pathway of PYL-PP2C interactions is conserved remain unknown. With the question of which PP2C member may be crucial for ABA response and whether the member could function through physical interaction with the PYL protein, we focused our study on a PP2C member that was found in a drought-treated RNA library. The expression of *PeHAB1* is induced by drought and ABA but not by high salt. This expression pattern is similar to *BolABI1* [30]. We compared the expression pattern of *PeHAB1* with several Clade A PP2Cs retrieved from *P*. *trichocarpa*, and found its accumulation pattern is similar to that of *Potri*.*018G060300*. Sequence alignment revealed that PeHAB1 and Potri.018G060300 exhibited 87.07% identity at the nucleic acid level and 92.16% identity at the amino acid level. Thus, they were supposed to be orthologs.

PP2C may interact with specific PYL members with or without ABA supplementation. For example, AtHAB1 interacted with AtPYL5 without the added ABA, and AtHAI3 exhibited strong interaction with AtPYL7 and weak interaction with AtHAI1 [31]. Here, we proved that PeHAB1 could interact with PePYL4 in the absence of ABA. However, PeHAB1 exhibited low-affinity with PePYL4 through the introduction of Trp420Ala mutation, indicating that the tryptophan residue located in the flap region of PeHAB1 is important for PYL4 interaction. Moreover, P124S mutation in the gate region of PePYL4 will destroy the affinity with PeHAB1 to a considerable extent, and this result is similar to that of AaPYL9 [25]. These results provided evidence that PeHAB1 may achieve its function of negative regulation in ABA signaling by interacting specifically with PYL(s) through gate-latch-lock mechanism.

ABA-agonists have the possibility to improve plant yield or any other properties under advance abiotic stress. Therefore, identification of ABA-agonists is important as a fundamental research [24]. We have previously identified several abiotic stress regulators in *P*. *euphratica*, certain of which are involved in ABA signaling (data not shown). However, core ABA signaling components have not been identified in *P*. *euphratica*. Characterization of *PP2C* genes may explore the ABA signaling study in this high abiotic stress tolerant tree.

## Materials and Methods

### Ethics approval

All *P*. *euphratica* seeds were collected in a public area where a natural *P*. *euphratica* population distributed along the Tarim River (40°51′N, 80°10′ E; Altitude, 972 m, 100–1500 m away from the river). *P*. *euphratica* is a protected species, but no permission for seed collection is required in China. The experiments for seed germination and seedling growth were conducted in the National Engineering Laboratory for Tree Breeding Lab (40°0′9′′ N, 80°20′15′′ E). This laboratory is owned and managed by Beijing Forestry University.

### Plant materials and treatments

Two-year-old *P*. *euphratica* seedlings were planted in individual pots (15 L) containing loam soil and placed in a greenhouse at Beijing Forestry University. *Arabidopsis* (Columbia ecotype) used for transformation were grown in 9-cm pots filled with customized soil mixture under cool-white light conditions (16 h light/8 h dark, 18,500 lux, 22°C, 70% relative humidity) until the flowers bloomed. For ABA and NaCl treatment, plants of *P*. *euphratica* were sprayed with 200 μM ABA solution or 300 μM NaCl. For drought treatment, *P*. *euphratica* were transferred from well-saturated soil to filter paper. Cold treatment was conducted by transferring young plants to a growth chamber set at 4°C under a 16 light/8 dark cycle. For all treatments, leaves were collected at the points of 0, 1, 3, 6, and 12 h and immediately frozen in liquid nitrogen. One-year-old seedlings of *P*. *tricorcarpa* were used to detect the expression levels of group A PP2C poplar candidates and the treatments were performed as described above.

### cDNA library construction

SMART cDNA library kit (Clontech, CA) was employed to construct a cDNA library for *P*. *euphratica*. Total RNA was extracted from drought-treated leaves collected at 3, 6, 9 h from two-year-old *P*. *euphratica* by using CTAB method [32]. After removing the contaminating genomic DNA with turbo DNA-free kit (Ambion), Oligo dT cellulose chromatography was used to enrich mRNA. After purification, cDNA was normalized and digested with *SfiI*, ligated into the *SfiI* predigested DNR-LIB vector, and transformed into *Escherichia coli* DH10B.The cDNA library was plated on LB plates with X-gal, isopropyl-D-thiogalactopyranoside and ampicillin. Approximately 1500 white colonies were randomly picked and sequenced.

### *PP2C* gene searching and *PeHAB1* isolation

Nucleotide homology searching was conducted by using BLAST program on Phytozome network. We define a match if a putative PP2C hit with an E-value ≤ 1e-10. Phytozome (http://phytozome.jgi.doe.gov/pz/portal.html) combined with Popgenie databases (http://popgenie.org/) were also used to search for *PP2C* genes in poplar. The full-length cDNA was isolated by polymerase chain reaction (PCR) with the primers 5’-ATGGAGGAGATGTATCCGG-3’ and 5’-TCATGTTTTGGTTTTGAACTTCC-3’. The phylogenetic tree was constructed by neighbor-joining bootstrap method (bootstrap analysis with 1,000 replicates). We used MEGA 6.0 software [33] based on multiple alignments of the amino acid sequences of 27 representative AtPP2Cs retrieved from the TAIR database (https://www.arabidopsis.org/) and all annotated poplar PP2Cs retrieved from Phytozome and Popgenie databases.

### Real-time quantitative reverse transcription PCR analysis

Quantitative PCR (qPCR) was performed to determine the gene expression level of ABA-related group A *PP2C* candidates from *P*. *trichocarpa* and *PeHAB1* isolated from the drought-treated cDNA library of *P*. *euphratica*. qPCR was conducted using a power SYBR Green PCR Kit (Applied Biosystems) with a StepOnePlus™ real-time PCR system [34]. The relative quantification value was calculated by the 2^−ΔΔCt^ method using respective *PtEF1a* and *PeActin* as internal control [35,36]. A multiple abiotic stress inducible gene *PeDREB2* was employed as experimental control in *PeHAB1* expression pattern analysis. All primers used in qPCR analysis are given in S1 Table. Each PCR assay was conducted for three biological replicates, and for each replicate, three technological replicates were repeated.

### Identification of PeHAB1-interacting proteins in poplar by yeast two-hybrid screening

The MoBiTec Grow'n'Glow two-hybrid system (MoBiTec, Goettingen, Germany) based on the Brent LexA Interaction Trap method was applied using the PeHAB1 as a bait [37]. Full-length *PeHAB1* were cloned into the pEG202 vector to fuse to the LexA DNA-binding domain. To moderate the inherent transactivation potential of full-length *PeHAB1*, we utilized the low sensitivity *Saccharomyces cerevisiae* strain EGY188, which contains two copies of the LexA operator upstream of the LEU2 reporter integrated in the yeast genome. pGNG1 vector was used as the reporter plasmid with a URA3 selectable marker and the GFP reporter gene, allowing for simple screening by UV transillumination. A pJG4-5 plasmid containing TRP1 selectable marker and SV40 unclear localization sequence inserted with *P*. *euphratica* cDNAs in the *EcoR*I and *Xho*I sites was employed in the screening. Plasmids were sequentially transformed into EGY188 by lithium acetate method. Colonies grown in DOBA-HIS-LEU-URA-TRP plate and showing green fluorescence when exposed to standard UV-light in darkroom were proposed to be putative colonies.

### Interaction confirmation

Potential positive transformants were confirmed by retransforming the yeast reporter strain with the cDNA of each potential candidate, streaking onto new DOBA-HIS-LEU-URA-TRP plates, and incubation for 24–72 h at 30°C. Colonies colonies were re-incubated in liquid medium and examined by a confocal microscope (Leica TCS SP5 II). The fusion of LexA and mouse p53 (AA 72–390) protein was served as positive bait control with the prey control vector pJG4-5-LTA which carries the gene for LTA, a protein known to interact with p53. GAL4-based two-hybrid assay was also used to verify interaction using pGBKT7-PeHAB1 as bait and pGADT7-PePYL4 as prey according to the Clontech’s protocol. For colonies containing different bait and prey plasmids, *β*-galactosidase activity was analyzed by colony-fit filter assay.

### Vector construction and plant transformation

Full-length *PeHAB1* cDNA was inserted into the pBI121 vector downstream of the 35S promoter of Cauliflower mosaic virus (CaMV) to construct the plasmids for transformation. The plasmid was introduced into *Agrobacterium tumefaciens* strain GV3101. Plants were then transformed by using floral-dip method [38]. The pBI121 vector was also transformed as a control. The presence of T-DNA in transgenic plants was confirmed by kanamycin selection as well as PCR detection of the *NPTII* gene and *HAB1* gene, as in a previous report [34].

### Germination assay

To determine the sensitivity of seedling inhibition established by ABA, a germination assay was performed. Approximately 100 seeds from each genotype were sterilized and sown on MS medium in triplicate. The medium contained 3% sucrose and 0.8% agar at pH 5.8 and was supplemented with different concentrations of ABA (0, 0.5, 1, and 3 μM ABA). Seeds were incubated at 4°C for 3 days before being placed at 22°C. The percentage of seeds that had germinated and developed fully green expanded cotyledons was determined, and germination was scored at the indicated time points. The germination and development for seeds in the medium with 0.75 μM ABA concentration were also recorded.

### Root growth assays

Root growth assay was also conducted to score ABA sensitivity. Sterilized seeds of T3 homozygous lines were first germinated and grown on MS agar medium for 5 days, and then transferred onto MS plates with or without 10 μM ABA. Plates were incubated in a vertical position in the growth chamber, and root length was investigated 5 days later.

### Transpiration rate and stomatal aperture measurements

For transpiration rate measurement, leaves were detached from 3-week-old plants, weighed immediately on a piece of weighing paper, and then subjected to air-drying (the aerial relative humidity was 40%, and the temperature was between 22 and 23°C). Weights were measured for 10 plants per line at the designated time points of 20, 40, and 60 min. The percentage of initial fresh weight was calculated at the indicated periods of time. Stomatal aperture was measured as in a previous work [34].

## Supporting Information

S1 Fig Sequence analysis of PePYL4. (A) cDNA and deduced amino acid Sequence of PePYL4. (B) Phylogenetic tree of PePYL4 and PYR/PYL/RCAR proteins from *Arabidopsis*. The tree was constructed by Neighbor–Joining method based on multiple alignments of the full-length amino acid sequences. The numbers next to each node give bootstrap values for 1000 replicates.
(TIF)

S1 Table Primer sequences use in qPCR analysis. (XLSX)
