# Supplementary figures and images for: A Putative PP2C-Encoding Gene Negatively Regulates ABA Signaling in Populus euphratica
Source: PLoS One. 2015 Oct 2;10(10):e0139466. doi: 10.1371/journal.pone.0139466 (PMC4592019; doi:10.1371/journal.pone.0139466)

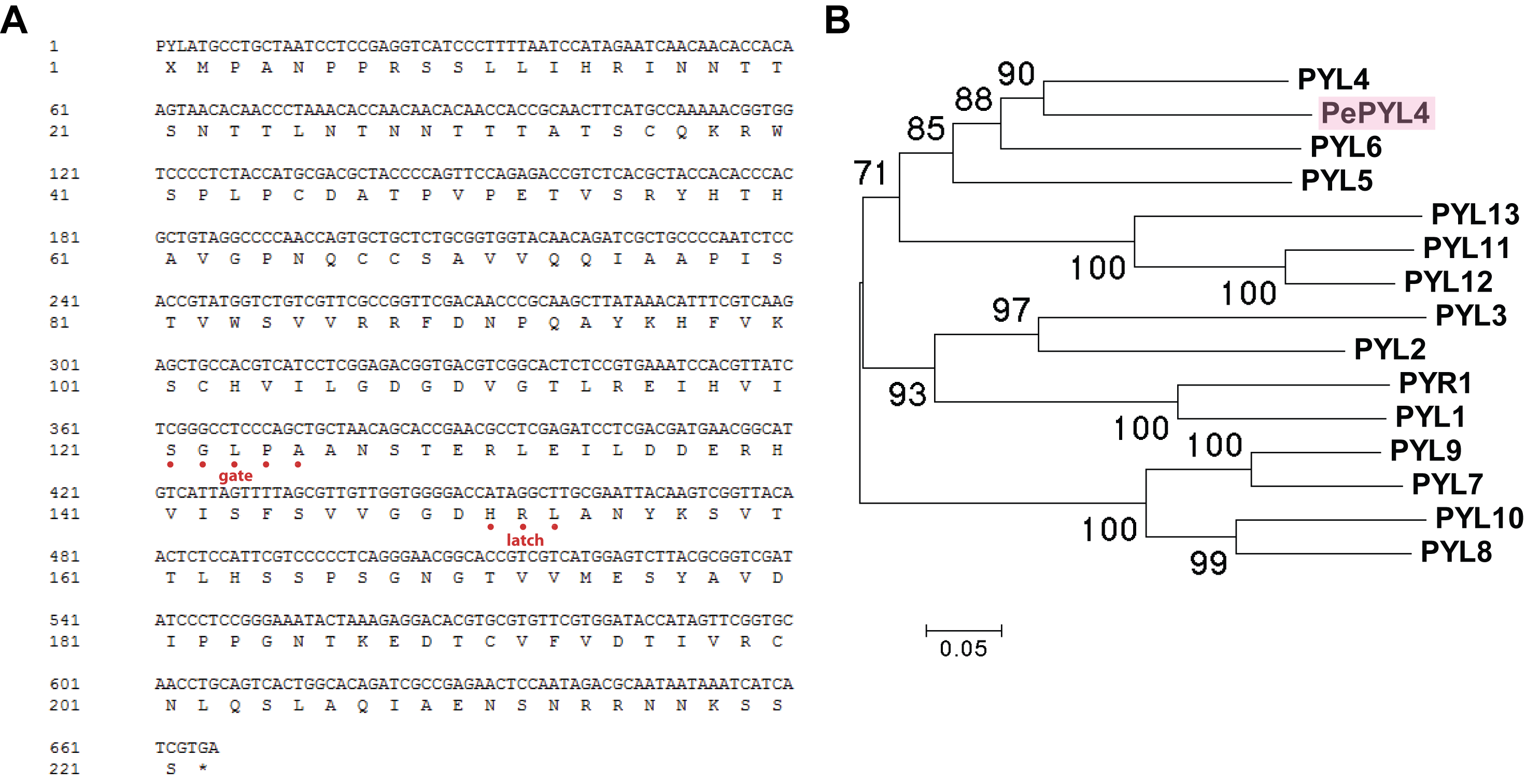

Supplement: S1 Fig — (A) cDNA and deduced amino acid Sequence of PePYL4. (B) Phylogenetic tree of PePYL4 and PYR/PYL/RCAR proteins from Arabidopsis. The tree was constructed by Neighbor–Joining method based on multiple alignments of the full-length amino acid sequences. The numbers next to each node give bootstrap values for 1000 replicates. (TIF) [file pone.0139466.s001.tif]
